# Sedentary Behavior and Physical Activity in Individuals With Bipolar Disorder: An Evidence Synthesis and Position Statement From the ISBD Nutrition and Exercise Task Force (NExT)

**DOI:** 10.1111/bdi.70164

**Published:** 2026-08-03

**Authors:** Beny Lafer, Lucas M. Neves, Davy Vancampfort, Brendon Stubbs, Ana Gonzalez‐Pinto, Andrew Nierenberg, Andrew T. Olagunju, Benjamin I. Goldstein, Eduard Vieta, Elena Koning, Fabiano A. Gomes, Gayatri Saraf, Jess G. Fiedorowicz, Lakshmi N. Yatham, Louisa Sylvia, Maj Vinberg, Manuel Gardea‐Resendez, Marco Solmi, Mark A. Frye, Michael Berk, Mohammad Alsuwaidan, Rodrigo B. Mansur, Roumen Milev, Simon Rosenbaum, Tamsyn Van Rheenen, Vicent Balanzá‐Martínez, Wolfgang Marx, Felipe Schuch, Elisa Brietzke

**Affiliations:** ^1^ Bipolar Disorder Program, Department of Psychiatry University of São Paulo Medical School São Paulo Brazil; ^2^ Department of Physical Education São Paulo State University (UNESP) Rio Claro Brazil; ^3^ Department of Rehabilitation Sciences KU Leuven Leuven Belgium; ^4^ Clinical Division of Social Psychiatry, Department of Psychiatry and Psychotherapy Medical University of Vienna Vienna Austria; ^5^ Comprehensive Center for Clinical Neurosciences and Mental Health (C3NMH) Medical University of Vienna Vienna Austria; ^6^ Psychological Medicine, Institute of Psychiatry, Psychology and Neuroscience (IoPPN) King's College London London UK; ^7^ Division of Psychology and Mental Health, Manchester Academic Health Science Centre University of Manchester Manchester UK; ^8^ Department of Psychiatry and Neurosciences, Charité Campus Mitte Charité – Universitätsmedizin Berlin Berlin Germany; ^9^ Hospital Universitario de Alava, BIOARABA, EHU, CIBERSAM Vitoria Spain; ^10^ Department of Psychiatry, Massachusetts General Hospital Harvard Medical School Boston Massachusetts USA; ^11^ Department of Psychiatry and Behavioral Neurosciences McMaster University/St Joseph's Healthcare Hamilton Hamilton ON Canada; ^12^ Department of Psychiatry and Behavioral Sciences University of Oklahoma Oklahoma City OK USA; ^13^ Discipline of Psychiatry Adelaide University Adelaide South Australia Australia; ^14^ Centre for Addiction and Mental Health, Department of Psychiatry University of Toronto Ontario Canada; ^15^ Institute of Neuroscience University of Barcelona, Hospital Clinic, IDIBAPS, CIBERSAM Barcelona Catalonia Spain; ^16^ Department of Psychiatry Queen's University School of Medicine Kingston Ontario Canada; ^17^ Department of Psychiatry and Behavioural Neurosciences McMaster University Hamilton Ontario Canada; ^18^ The Ottawa Hospital and Ottawa Hospital Research Institute Ottawa Ontario Canada; ^19^ Department of Psychiatry University of British Columbia Vancouver Canada; ^20^ The Early Multimodular Prevention and Intervention Research Institution (EMPIRI), Mental Health Centre Northern Zealand Denmark; ^21^ Copenhagen University Hospital – Mental Health Services CPH Copenhagen Denmark; ^22^ Department of Clinical Medicine, Faculty of Health and Medical Sciences University of Copenhagen Copenhagen Denmark; ^23^ Department of Psychiatry Universidad Autonoma de Nuevo Leon Monterrey Mexico; ^24^ Department of Psychiatry, Faculty of Medicine University of Ottawa Ontario Canada; ^25^ Department of Child and Adolescent Psychiatry Charité Universitätsmedizin Berlin Germany; ^26^ Department of Psychiatry & Psychology Mayo Clinic College of Medicine Rochester Minnesota USA; ^27^ Deakin Institute for Mental and Physical Health and Clinical Translation (Deakin IMPACT), School of Medicine Deakin University Geelong Australia; ^28^ Mental Health Drug and Alcohol Services, Barwon Health University Hospital Geelong Geelong Australia; ^29^ Orygen, The National Centre of Excellence in Youth Mental Health, Centre for Youth Mental Health, Florey Institute for Neuroscience and Mental Health and the Department of Psychiatry The University of Melbourne Melbourne Australia; ^30^ MindWell Center Kuwait City Kuwait; ^31^ Department of Psychiatry University of Toronto Toronto Ontario Canada; ^32^ Department of Psychiatry, Centre for Neuroscience Studies Queen's University, Providence Care Kingston Ontario Canada; ^33^ Discipline of Psychiatry and Mental Health, School of Clinical Medicine, Faculty of Medicine and Health UNSW Sydney Sydney Australia; ^34^ Department of Psychiatry, Faculty of Medicine, Dentistry and Health Sciences University of Melbourne Carlton Australia; ^35^ Centre for Mental Health and Brain Sciences Swinburne University Hawthorn Victoria Australia; ^36^ Teaching Unit of Psychiatry, Department of Medicine University of Valencia Valencia Spain; ^37^ Deakin Institute for Mental and Physical Health and Clinical Translation, Food & Mood Centre, School of Medicine, Barwon Health Deakin University Geelong Australia; ^38^ Institute for Mental and Physical Health and Clinical Translation (IMPACT) and Institute for Physical Activity and Nutrition (IPAN) Deakin University Geelong Australia

**Keywords:** bipolar disorder, depression, exercise, lifestyle medicine, mania, mood, physical activity mental health, sedentary behavior

## Abstract

**Background:**

Higher time in sedentary behavior and insufficient physical activity are very common in bipolar disorder (BD). Whereas physical activity and exercise offer significant benefits across mental health, brain health, and heart health in BD. The International Society for Bipolar Disorders Nutrition and Exercise Task Force (NExT) synthesized evidence to evaluate the impacts of sedentary behavior, physical activity, and exercise on illness course, physical health, and treatment outcomes.

**Methods:**

A best‐evidence synthesis was conducted using a question‐and‐answer format. We searched PsycINFO, SPORTDiscus, Embase, PubMed, and Web of Science from inception to March 1st, 2026, supplemented by reference lists and existing guidelines. Search terms included combinations of physical activity, exercise, sedentary behavior, and bipolar disorder‐related terms.

**Results:**

Observational studies show that individuals with BD exhibit significantly higher sedentary behavior and lower physical activity compared to the general population, with notable discrepancies between self‐reported and objective measurements. Evidence on physical activity's protective role against BD onset is mixed, with some prospective and Mendelian randomization studies suggesting reduced risk, while others report bidirectional or null associations. Preliminary findings from clinical trials indicate that exercise improves overall functioning, reduces mood episode frequency and hospitalizations, and alleviates depressive symptoms, with one pilot study showing an 82% antidepressant response rate. Exercise also shows potential to reduce anxiety and improve sleep, though robust data are limited.

**Conclusions:**

Despite limited high‐quality evidence, physical activity and exercise demonstrate promising physical and mental health benefits for individuals with BD. Future research should prioritize multicenter randomized controlled trials, enhanced assessment tools, and accessible, tailored programs to integrate physical activity into routine BD management.

## 1 Introduction

Bipolar disorder (BD) is associated with elevated rates of obesity, metabolic syndrome, and early onset cardiovascular disease [1, 2, 3]. Individuals with BD face a 76% higher risk of cardiovascular morbidity and mortality compared to the general population [4]. The drivers of this excess burden are multifactorial, including inequities in health care access [5], adverse metabolic effects of pharmacotherapy [6], and poor lifestyle factors such as unhealthy dietary patterns, prolonged sedentary behavior (SB), low levels of physical activity (PA), and limited engagement in exercise [7, 8, 9].

It is well established that people with BD struggle to meet the public health recommendations for PA, and spend a disproportionally high amount of time in SB [7, 10]. Although related, PA and SB are different constructs [11, 12, 13]. This position statement adopts the definitions outlined in the World Health Organization (WHO) guidelines on PA and SB [11]: SB refers to any waking behavior characterized by energy expenditure below 1.5 metabolic equivalents of task (MET), typically while seated or reclined. PA encompasses any body movement that results in energy expenditure (≥ 1.5 MET). Exercise is a structured subset of PA and generally has the aim to improve or maintain physical fitness (e.g., cardiovascular endurance, muscular strength, body composition). Two additional dimensions are important when examining the health effects of PA: its intensity (e.g., light, moderate, and vigorous), and domain (e.g., leisure, occupational, household, transport).

On an absolute scale, light intensity PA refers to activities of 1.5 but less than 3 METs (e.g., walking, bathing, washing dishes), moderate intensity PA includes activities ranges from 3 to 5.9 MET (e.g., brisk walking, recreational swimming, carrying boxes), and vigorous intensity PA comprises activities of 6 or more MET (e.g., jogging or running, swimming laps, heavy yard work) [14, 15]. These activities may occur in different domains: leisure time activities, involving activities on non‐work undertaken for enjoyment and personal fulfillment (e.g., sports participation, exercise conditioning, or recreational activities), occupational activities, encompassing tasks performed during paid or voluntary work (e.g., bakery, manual labor, construction work), household activities, consisting of domestic tasks conducted at home (e.g., cleaning, childcare, gardening) and transport/commuting activities, related to travel to and from locations (e.g., walking, cycling) [11]. Regardless of the domain, the WHO recommends that adults should accumulate 150–300 min/week of moderate intensity, or 75–150 min of vigorous intensity, or a combination of both, while children and adolescents should engage in at least 60 min of moderate‐to‐vigorous daily. WHO guidelines also emphasize regular muscle strengthening activities for all ages (5–17 years; 18–64 years; over 65 years), such as resistance training or body‐weight exercises [11].

Unlike PA, the WHO guidelines [11] do not provide explicit thresholds for SB. Instead, they broadly recommend minimizing sedentary time across the lifespan, notably prolonged bouts (e.g., more than 2 h), and total volume of SB. However, evidence suggests that spending more than 6 to 8 h per day sedentary increases the risks of cardiovascular diseases [16, 17] and all‐cause mortality in the general population [18]. Prolonged sedentary time is also associated with a range of mental health conditions, including depression, anxiety, schizophrenia, and BD [19, 20, 21].

PA and exercise are consistently associated with physical and transdiagnostic mental health benefits. PA lowers the risk of premature mortality and a wide spectrum of chronic diseases [22], including metabolic syndrome [23], type 2 diabetes [24], cardiovascular disease [25], metabolic associated fatty liver disease [26], overweight/obesity [27], dementia [28], depression [29] and anxiety [30]. Additionally, exercise has demonstrated efficacy as an adjunctive treatment for mental disorders [31, 32, 33, 34, 35, 36, 37], and is already included in many mental health guidelines [38, 39, 40].

Despite these benefits, the current guidelines for BD remain limited in their practical recommendations for SB, PA, and exercise. The 2018 guidelines from the Canadian Network for Mood and Anxiety Treatments (CANMAT) and the International Society for Bipolar Disorders (ISBD) emphasize the significance of managing PA in conjunction with psychological or pharmacological interventions, with an additional focus on mitigating the risk of cognitive decline [41]. The National Institute for Health and Care Excellence (NICE) Guidelines for BD assessment and management recommend at least an annual assessment of PA levels, but do not provide specific guidance on SB, PA, and exercise [42]. Broader mental health guidelines have begun incorporating lifestyle interventions, but their focus often lies outside BD. For example, the CANMAT 2024 guidelines [40] address lifestyle interventions in the context of perinatal mood, anxiety, and related disorders (PMADs), particularly postpartum depression [43, 44, 45, 46, 47, 48, 49, 50, 51]. Similarly, the Royal Australian and New Zealand College of Psychiatrists (ANZCP) guidelines describe the potential benefits of exercise in BD, such as improved quality of life and antidepressant effects, but do not address SB and PA [52]. The World Federation of Societies of Biological Psychiatry (WFSBP) and Australasian Society of Lifestyle Medicine (ASLM) guidelines are targeted toward major depressive disorder (MDD) [39], while the European Psychiatric Association (EPA) guidelines [38] provide no BD‐specific recommendation for SB, PA, and exercise. Collectively, existing guidelines do not offer comprehensive BD‐specific recommendations in these areas.

Given the well‐documented benefits of PA and exercise for physical and mental health in the general population and the limited guidance for BD, the International Society for Bipolar Disorders (ISBD) established the “Nutrition and Exercise Task Force” (NExT). NExT brings together a multidisciplinary team of psychiatrists, psychologists, physiotherapists, professionals of physical education, dietitians, and other experts. Its overarching goals are to produce evidence‐based position papers on nutrition and exercise in BD; to support psychiatrists and allied professionals by providing guidance on the role of lifestyle factors in illness risk, course, treatment response, and medical comorbidities, and to advance understanding and advocate for the routine use of evidence‐based lifestyle approaches as potential adjunctive treatments. NExT also aims to foster research by broadening perspectives on lifestyle approaches, stimulating the generation of new knowledge in this field.

The specific aims of this consensus paper are:

1. To critically appraise the literature related to SB, PA, and exercise in BD.
2. To review the evidence on the role of exercise in relation to illness risk, course, treatment response, medical comorbidities, and its potential as an adjunctive treatment for acute episodes and long‐term prophylaxis
3. To provide researchers with a comprehensive overview of PA, SB, and exercise and encourage further investigation and innovation in this area.

To address these aims, we conducted a best evidence synthesis in a question‐and‐answer format to address these crucial issues. Where BD specific evidence was lacking, we drew upon data from related mental disorders (e.g., MDD) or from the general population as proxies, in order to identify critical gaps and highlight priorities for further research.

## 2 Methods

The task force identified nine critical questions relevant for clinical care based on expert opinion through discussion among its members. To answer these questions, literature searches were conducted for relevant studies published in English using searches of electronic databases (PsycINFO, SPORTDiscus, Embase, PubMed, and Web of Science databases) from inception to March 1st, 2026, an inspection of references list, and a review of other guidelines and major reports for people with mood disorders and BD. We also reviewed recent consensus statements and guidelines on PA and SB for the general population [11, 53]. General searches were conducted independently by two reviewers, with discrepancies resolved through consensus. Filters included English‐language studies and human participants. Gray literature, such as conference abstracts, was excluded to focus on peer‐reviewed publications. Eligible studies included observational, interventional, randomized controlled trials (RCTs), review and meta‐analysis involving individuals with BD, focusing on SB, PA, or exercise outcomes. Studies were excluded if they lacked BD‐specific data or were not published in English.

The literature search was conducted using the following terms: (physical activity OR exercise OR sedentary behavior) AND (bipolar OR bipolar disorder OR Mania OR Bipolar Affective Disorders OR Manic‐Depressive Psychosis).

Considering the recommendation regarding PA made by the WHO is expressed in minutes per week [11], NExT adopted the criteria to express PA in minutes per week (min) and SB in minutes per day (min). Due to heterogeneity in study designs and measurement methods, a narrative best‐evidence synthesis was employed, prioritizing studies with objective PA/SB measures and higher methodological quality.

## 3 Results

To address the nine questions of NExT, we identified studies involving individuals with BD, encompassing SB, PA, or exercise outcomes according to the following designs: Twenty‐eight observational studies [54, 55, 56, 57, 58, 59, 60, 61, 62, 63, 64, 65, 66, 67, 68, 69, 70, 71, 72, 73, 74, 75, 76, 77, 78, 79, 80, 81], 11 interventional studies [82, 83, 84, 85, 86, 87, 88, 89, 90, 91, 92, 93], four study protocols [94, 95, 96, 97], three reviews [89, 98, 99], 11 systematic reviews [4, 10, 46, 54, 100, 101, 102, 103, 104, 105, 106], and three meta‐reviews or umbrella reviews [32, 36, 37]. Additionally, we identified studies with other mental disorder populations that are relevant to our research questions, categorized by the following designs: seven observational studies [19, 21, 107, 108, 109, 110, 111], one interventional study [112], one study protocol [113], six reviews [114, 115, 116, 117, 118, 119], 10 systematic reviews [33, 120, 121, 122, 123, 124, 125, 126, 127, 128], three meta‐reviews or umbrella reviews [36, 129, 130], and one consensus study [131].

### 3.1 How Much PA and SB Do People With BD Engage in Relative to the General Population?

Vancampfort et al. performed a systematic review and meta‐analysis, summarizing studies from inception until February 1, 2016, which identified six studies concerning PA and bipolar disorders (279 individuals total—objective or subjective measure). The common metric across included studies was total PA, which encompasses all intensities of PA per day (e.g., low intensity, moderate intensity, and vigorous intensity), with durations varying from 98.4 to 287.6 min, with estimates of 98.4 min [78], 127.6 min [63], 137.0 min [78], 229 min [60], 285.7 min [118], and 287.6 min [119]. Regarding moderate‐intensity PA, the durations varying from 8.4 to 168.5 min, with estimates of 8.4 min [112], 28.7 min [78], 80.1 min [76], and 168.5 min [77], while the duration of vigorous‐intensity PA ranges from 5.8 to 56.3 min, with estimates of 4.9 min [112], 5.8 min [78], 39.7 min [77], and 56.3 min [76]. Important to highlight that even the value of the summarized studies, the lack of current mood states during data collection may have introduced bias into the results.

Considering mood states, a recent study by Monteiro et al. [66] evaluated depressive symptoms using the Hamilton Depression Rating Scale (HAM‐D) and manic symptoms using the Young Mania Rating Scale (YMRS) at the beginning of a PA data collection. The study demonstrated that subjects with BD in the depressive phase (*n* = 15; HAM‐D = 13.6 ± 5.8 points; YMRS = 2.9 ± 2.7 points) exhibited a significantly lower duration of moderate‐intensity PA, with a duration of 102 ± 89 min, compared to those in the manic phase (*n* = 11; HAM‐D = 6.9 ± 4.5 points; YMRS = 11.8 ± 2.1 points), who engaged in moderate‐intensity PA with a duration of 206 ± 144 min.

More recent studies employing objective measures have reported weekly averages ranging from 98 to 238 min of moderate‐intensity PA, with estimates of 98 min [55, 60] 155 min [56], and 238 min [58]. These values suggest that many individuals with BD fall short of the WHO guidelines of 150–300 min of moderate‐to‐vigorous PA for adults, though some may approach the lower threshold. Despite the absence of studies documenting the comparing PA of adolescents or older adults with BD with their peers without BD, Jeweel et al. demonstrated that adolescents with BD were significantly less likely to report engaging in regular PA compared to the control group [61].

Regarding SB, the meta‐analysis by Vancampfort et al. [10] estimated that the total daily time spent in SB was 613 min. Research employing objective accelerometry [60] confirmed these findings, showing that people with BD spend significantly more time sedentary compared to healthy controls (812 min vs. 539 min).

As previously mentioned, various factors can influence the accumulation of PA and SB time, including the data collection strategy (objective or subjective), the intensity of the PA (total PA, moderate PA, or vigorous PA), and mood state. Furthermore, for BD, we emphasize the geographic factors (country) and the characteristics of subjects with BD, distinguishing between outpatient and inpatient statuses, as outlined in Table 1. A prior study indicated that the “built environment”, encompassing the surroundings in which individuals reside and work, influences PA [132]. Attributes of the built environment, such as destination accessibility, connectivity, walking and cycling infrastructure, safety, and esthetics, are positively correlated with physical activity [133]. Furthermore, meteorological variables and social contexts exert influences on PA and SB [134]. Consequently, research on PA and SB in individuals with bipolar disorders requires a more detailed examination of these factors.

**TABLE 1:** Factors to be considered in the interpretation of results from studies evaluating PA and SB in individuals with BD.

Factor	Description
Mood state	Individuals in mood states (mania or depressive episodes) with higher levels of activation may have higher PA levels both in subjective and objective assessments [79]
	PA ‐ Individuals in the manic phase engaged in moderate‐vigorous PA (*p* < 0.05) in a greater duration (206 min) compared to those in the euthymic phase (125 min) or the depressive phase (102 min) [66]
	SB ‐ Individuals in the depressive phase (428 min) engaged in SB (*p* > 0.05) for a greater duration than manic phase (321 min) compared to those in the euthymic phase (368 min) [66]
Circadian rhythm desynchronization	Peak in activity during the morning, decline in activity during the night and increased variability when compared BD I with BD II [70, 74]
	**Duration of moderate PA**
Geographic factors	USA (98 min) [60]
	Brazil (113 min) [56]
	Brazil (137 min) [66]
	Belgium (168 min) [77]
	Denmark (238 min) [58]
	Poland (465 min) [59]
	**Duration of SB**
	Brazil (377 min) [66]
	Brazil (480 min) [56]
	Belgium (613 min) [10]
	Poland (630 min) [59]
	USA (812 min) [60]
Source of the sample (outpatient vs. inpatient)	Mixed sample of outpatients and inpatients (543 min) [10]
	Outpatient (647 min) individuals showed similar levels of daily SB [10]
Community vs. inpatient	Patients in community settings had a lower level of PA (128 min), compared to those in inpatient settings (286 min) [10]
Intensity of the PA	PA at a light intensity (1071 min), vigorous intensity PA (161 min) [10]
	Light intensity PA (2063 min) and vigorous intensity (0 min) [56]

Abbreviations: BD, bipolar disorders; PA, physical activity; SB, sedentary behavior.

The NExT Task Force identified several critical issues for interpretation (Table 1). The first one relates to the variability in psychomotor activity and energy, which is a hallmark feature of BD, noted since Kraepelin's early description [135]. Some authors argue [136] that fluctuation in the energy levels should be considered a primary symptomatic dimension of BD. However, in DSM‐5 [137], mood alteration remains the core diagnostic criterion for a diagnosis of a depressive or a manic/hypomanic episode, with increased or decreased psychomotor activity and energy listed as secondary features. While in the general population, PA levels are not strongly influenced by mood fluctuations, it may not be the case for individuals with BD. Some researchers [102] have therefore proposed the construct of “Activation”, integrating DSM‐5 symptoms dimensions observable at both the behavioral (psychomotor agitation/retardation) and subjective levels (levels of energy).

In the same way, it is well known that individuals with BD often experience substantial intra‐day and diurnal variability in energy levels. SB can be conceptualized not only as total time spent sedentary but also in terms of its pattern, including timing of the day, duration, and frequency of bouts and breaks [138]. For example, Shou et al. [70] investigated motor activity patterns among euthymic persons with different subtypes of mood disorders (BD type I or II) compared to unaffected controls. After adjusting for the influence of age, gender, body mass index, and medication usage, BD I participants showed lower median levels of activity intensity in the latter part of the day and greater variability in the afternoon compared with controls. In contrast, Tanaka et al. [74] demonstrated individuals with BD exhibit a pronounced morning activity peak followed by a steep decline in activity during the night, suggesting distinctive circadian‐related activity profiles; such patterns may provide additional objective markers of illness course or subtype, complementing symptom‐based assessments.

In summary, individuals with BD consistently spend more time in SB and often fail to meet recommended levels of PA compared with the general population. Furthermore, their PA and SB patterns appear closely tied to fluctuations in energy and psychomotor activity, which vary with illness stage and circadian rhythms. These findings highlight the need for research that goes beyond total time measures to capture the temporal patterns and variability of activity, which may have diagnostic and prognostic value in BD.

### 3.2 What Is the Evidence of the Efficacy of Exercise in Bipolar Depression?

Currently, we found four randomized controlled trial study protocols [94, 95, 96, 97, 113] being carried out involving BD and exercise, using strength exercise, high‐intensity interval training (HIIT), aerobic exercise, or aerobic + strength exercise protocols. Additionally, studies with yoga, a holistic multi‐dimensional health and well‐being system, focused on the mind and its functions with multi‐component mind–body practices, including physical postures and movement, breathing exercises, relaxation, and mindfulness and meditation [131] were identified in subjects with BD. Although it does not correspond to direct intervention through physical exercise but focuses on behavioral modification strategies for exercise practice, counseling interventions aimed at enhancing aerobic fitness [82] or a combination of nutrition, exercise, and wellness [90] were identified.

Two studies evaluated the acute response (a single session that induces immediate, temporary physiological alterations) of exercise in BD. The research conducted by Shickh and collaborators [88], which examined exercise preference and tolerance in 107 youths with BD, revealed that exercise preference was significantly associated with lower cardiorespiratory fitness and higher perceived exertion, while higher exercise preference was associated with a history of psychiatric hospitalization. The lower exercise tolerance in youth with BD was significantly associated with female sex, elevated perceived exertion, and non‐Caucasian ethnicity. A recent study [87] that included eight BD patients and eight controls demonstrated that continuous moderate‐intensity exercise and high‐intensity interval training (HIIT) produced beneficial autonomic and mood responses in patients with bipolar disorder and healthy controls.

Considering the number of study protocols available on the relationship between exercise and BD, indicating that more evidence may emerge in the near future. We highlight that to better understand the impact of exercise on BD, it's important to consider the biopsychosocial model [36, 116], which includes biological benefits such as neurotransmitter systems, decreased inflammation, and neurogenesis, psychological benefits such as self‐esteem and self‐efficacy, and social benefits such as significant interactions. Objective assessments of physical fitness, including muscular strength and cardiorespiratory fitness, can strengthen the link between physical fitness and symptoms of BD.

Regarding the available studies with BD samples, these are predominantly pilot studies. The pilot study of Weinstock et al. [93] showed yoga intervention by 10 weeks results in significant improvement in depression compared to control condition. Other pilot studies involving qigong/tai chi interventions [86] or concentrate on self‐management sessions, symptom control, healthy habits, and provider engagement [83], demonstrated feasibility for individuals with BD. The pilot trial conducted by Lafer et al. [84], examined the impact of 12 weeks, three times per week of exercise (aerobics and strength exercises) intervention on individuals with BD. The study involved 15 subjects with BD, utilized the Montgomery Åsberg Depression Rating Scale (MADRS), and demonstrated that 82% of participants exhibited an antidepressant response, characterized by a reduction exceeding 50% in depressive symptoms. Additionally, 45% of these patients met the criteria for complete remission. The baseline MADRS score observed was 23.6 ± 8.3 points, and post intervention, the score decreased to 10.2 ± 4.8 points.

Different meta‐analyses summarized and showed the antidepressant effect of PA on MDD [33, 35, 114, 125, 127], but on BD we identified only one. Li and colleagues [101] summarized the effectiveness of exercise interventions on depressive and manic symptoms in patients with BD. Of the seven studies included, only four [83, 86, 91, 93], already cited here, were published in the databases considered for this research, while three additional studies are accessible via Wanfang Data, a prominent Chinese academic information service. The authors demonstrated a significant effect (*p* < 0.05) of these interventions on depressive symptoms (effect size = 0.63, 95% CI: −1.11 to −0.14), and not a significant effect on manic symptoms (*p* > 0.05).

In summary, it is well documented that exercise is efficacious in treating MDD and reducing depressive symptoms severity. Different forms of exercise, such as aerobics (walking, running), strength, and yoga, have an antidepressant effect. Children and adolescents, as well as adults and the elderly, can benefit from the antidepressant effect of exercise. Different biases are identified in all meta‐analyses, but after sub‐analysis, considering most of these biases, the antidepressant effect persists.

Given the extant evidence, exercise has the potential to reduce depressive symptoms in people with BD.

### 3.3 Why Is Promoting PA Critical for Physical Health and Psychosocial Outcomes in People With BD?

The benefits of PA are established in the WHO guidelines, supported by strong evidence (“A” certainty) that PA and SB influence multiple health outcomes. Specifically, regular PA and reduced SB lower the risk of adiposity‐related outcomes such as overweight, obesity, or high body mass index, diabetes mellitus, cardiovascular disorders, osteoporosis, metabolic syndrome, and obesity [11, 139, 140]. Additionally, PA promotes cardiorespiratory fitness and muscular strength, both of which are robust predictors of morbidity and mortality across populations [141, 142, 143].

Considering that people with BD consistently show to engage in lower time of PA [55, 56, 58] and higher time of SB [56, 60, 77], as a result, they face disproportionately high risk for developing metabolic syndrome [106], diabetes mellitus [104], osteoporosis [62], obesity or metabolic syndrome [144], metabolic associated fatty liver disease and cardiovascular disorders [122]. Weight gain, which is related to most diseases mentioned, often occurs with medications such as antidepressants and antipsychotics [109, 120, 128]. As a consequence, epidemiological studies further confirm excess mortality rates from natural causes, especially cardiovascular and cerebrovascular disease in people with BD [4]. Despite this elevated risk profile, research [130] specifically examining whether increasing PA or decreasing SB can mitigate these medical comorbidities in BD remains limited. This gap hinders the development of evidence‐based, disorder‐specific recommendations for PA and SB in BD care.

Beyond general health, PA also confers psychosocial benefits. Evidence from other mental disorders indicates that PA can improve self‐esteem, reduce internalized stigma, and enhance self‐efficacy, defined as one's belief in one's own abilities to execute a task and achieve predetermined objectives [62, 116, 145], such psychosocial outcomes may be particularly important for individuals with BD, who often face challenges of social isolation and diminished self‐efficacy, both of which are negatively associated with lower PA participation [99]. Enhancing self‐efficacy through engagement in PA may not only foster sustained PA but also improve illness self‐management, social connectedness, and overall quality of life. These psychosocial dimensions may therefore serve as critical mechanisms by which PA contributes to improved outcomes in BD, complementing its well‐documented physical health effects. McCartan et al. [98] summarized effective strategies to promote PA in BD based on a systematic review of qualitative evidence. They identified the importance of clinical stability in BD, consideration of gender differences, the influence of stigma, and financial constraints for people with BD participating in PA. Additionally, they identified practical challenges such as adverse weather conditions, transportation issues, and insecurity that affect regular PA participation. The absence of skill, expertise, or confidence also was a factor hampering engagement in PA by people with BD. In addition, environmental variables, including the built environment, urbanicity and green space availability, affect both BD and PA. Ultimately, technology‐driven PA monitoring may enlighten individuals with BD about their PA time.

In summary, people with BD are at a heightened risk of a wide range of other medical comorbidities and excess mortality. While the general health benefits of PA are firmly established in the general population, and its psychosocial benefits are increasingly recognized in mental health contexts, disorder‐specific evidence in BD is still scarce. Future research should prioritize longitudinal and interventional studies to clarify how PA interventions, and reductions in SB, can simultaneously address the medical and psychosocial vulnerabilities of this population.

### 3.4 Is There a Relationship Between PA and the Course of Illness and Outcomes of BD?

Evidence investigating whether PA affects the course and progression of BD is still limited. One of the few available studies, conducted by Ng et al. [67], provides preliminary insights. Their findings suggest that patients who remained physically active during their treatment exhibited higher levels of overall functioning across several domains, including autonomy, occupational functioning, cognitive performance, financial management, interpersonal relationships, and leisure engagement. Conversely, reduced activity was associated with greater frequency of mood episodes and increased likelihood of psychiatric hospitalizations [67]. These observations align with broader evidence linking behavioral activation and structured daily routines to improved outcomes in BD, highlighting the potential of PA as a mood stabilizing, and potentially cognition‐enhancing factor within illness management. Preliminary findings [64] further suggest that maintaining PA may serve a protective role against relapses, while declines in PA participation could act as an early warning sign of clinical deterioration. Despite these promising indications, the evidence base remains sparse. A pilot study integrating nutrition, exercise, and wellness [91], demonstrated intervention efficacy, evidenced by increased weekly exercise duration and reduced depression and illness severity throughout the study period. Few studies have directly evaluated the longitudinal effects of PA or SB on BD outcomes, and available data are largely limited to small cohorts or secondary analyses. Moreover, causal inference is complicated by the bidirectional nature of PA and illness course, that is, mood symptoms (e.g., depression, psychomotor slowing, or manic overactivity) can significantly influence PA levels, while conversely, regular PA may enhance resilience, support social integration, and mitigate comorbid risks, thereby potentially affecting relapse and recovery trajectories. As such, preliminary evidence suggests that PA may improve functioning in multiple domains, and reducing PA may result in an increased frequency of mood episodes and psychiatric hospitalizations.

In summary, early findings suggest that greater PA engagement may support functional recovery and illness stability in BD, while reduced activity may signal or contribute to illness exacerbation. However, the paucity of rigorous longitudinal and interventional studies limits the strength of current conclusions. As outlined in Table 2, potential pathways by which PA and SB might influence BD include effects on functioning, relapse risk, comorbidity burden, and hospitalization. Future research should prioritize prospective designs and mechanistic studies to clarify these relationships and determine whether PA interventions can be integrated into relapse prevention and functional rehabilitation strategies for BD.

**TABLE 2:** Potential benefits of physical activity in the clinical course of BD.

Dimension of benefit	Outcomes
Psychiatric symptoms severity	Anxiety symptoms [67]
	Depressive symptoms [67]
	Insomnia [67]
Course of illness	Reduction in risk of mood episodes [64]
	Reduction in risk of hospitalization [64]
Metabolic factors	BMI [64]
General functioning	Functioning [64, 91]
	Cognition [64]

Abbreviation: BMI, body mass index.

### 3.5 What Is the Impact of Exercise on Common Psychiatric Comorbidities in BD (Anxiety, Suicidal Ideation, Substance Abuse Disorders)?

Emerging evidence suggests that PA may play an important role in reducing anxiety and stress symptoms, as well as in promoting better sleep regulation among individuals with BD. A retrospective cohort study of BD patients admitted to a private psychiatric clinic [67] found that those who participated in a structured walking group during hospitalization reported significantly lower scores on the Depression Anxiety Stress Scales compared to non‐participants. This finding highlights the potential short‐term psychological benefits of incorporating even low‐intensity, accessible forms of PA, such as walking—into inpatient care routines. Complementing these findings, Melo et al. [64] conducted a prospective study, in which patients with BD had their PA levels monitored alongside monthly reviews of medical records. Results indicated that patients who engaged in regular PA experienced decreased anxiety symptoms and fewer complaints of insomnia, suggesting a beneficial role for PA in daily symptom management. The already mentioned meta‐analysis of Li and colleagues [101] also summarized the effectiveness of exercise interventions on anxiety symptoms in patients with BD, demonstrating a significant effect (*p* < 0.05) on anxiety symptoms (effect size = −0.70, 95% CI: −1.26 to −0.15). About sleep outcomes, previous literature [89] suggested that exercise may also stabilize circadian rhythms and improve sleep. Given that circadian rhythm disruption is a hallmark feature of BD and a well‐recognized precipitant of mood episodes, the potential of PA to support sleep regulation and rhythm stability is of particular clinical importance in this population.

Together, these findings suggest that regular PA participation may alleviate anxiety, reduce stress, and improve sleep among individuals with BD. While the available evidence is still limited in scale and design (e.g., retrospective, or small prospective studies), the consistency across studies and alignment with circadian biology models strengthens the plausibility of these effects. Future research should aim to determine the optimal type, intensity, and timing of PA to maximize benefits for anxiety reduction and sleep regulation in BD, while also evaluating whether these improvements translate into greater mood stability and relapse prevention.

### 3.6 What Is the Relationship of SB and PA and Cognition of Individuals With BD?

In the general population, physical fitness measures are associated with cognitive outcomes [146, 147]. A study of 22,699 MDD patients and 1475 BD patients [57] also showed that maximal handgrip strength, an index of muscular strength, was associated with better visual memory, response time, prospective memory, and reasoning performance in BD patients and better performance on visual memory, reasoning, number memory, and prospective memory performance in those with MDD. In addition, a study with adolescents with BD [85] showed that a single session of moderate‐intensity aerobic exercise (20 min of cycling at approximately 70% of maximal heart rate) affected neural responses during an attention and inhibition task. In this study, neuroimaging findings revealed that before exercise, those with BD showed atypical patterns of activation and deactivation, particularly in the ventral prefrontal cortex, amygdala, hippocampus, and anterior cingulate cortex, which correlated with depressive and manic symptoms. After exercise, these pre‐exercise symptom–brain activation relationships were no longer evident, and task performance matched that of control subjects. Though no behavioral effect was observed, the study suggests that even a single bout of aerobic exercise can transiently normalize neural activity usually associated with impaired executive control in adolescents with BD, shedding light on potential mechanisms and therapeutic implications.

Three different meta‐analyses [124, 126, 129] of unipolar depression have summarized the effect of structured exercise in cognition, where exercise was shown to improve attention and information‐processing speed [124], a composite index of inhibitory control, cognitive flexibility, reasoning/planning, verbal learning and memory, visual learning and memory [129], and overall cognitive function and the sub‐domains of memory and executive function [126], respectively. In BD specifically, despite growing evidence of the efficacy of lifestyle interventions for improving depressive symptoms and functioning [103], cognition remains a less investigated outcome. For example, a recent systematic review of randomized clinical trials [100] found only seven lifestyle‐based interventions targeting cognition in BD, and none included PA or exercise.

As with PA and exercise, very few studies have examined the association of SB with cognition in BD, but the evidence arising from existing studies suggests the association is nuanced. Indeed, one study [68] using a large sample from the UK Biobank showed that self‐reported mentally‐passive SB (tv viewing), and counter‐intuitively, higher self‐reported PA, were associated with poorer cognition in people with BD and healthy controls. In that study, mentally‐active SB (leisure‐time computer use) was associated with better cognition and was also found to moderate putative age‐related cognitive decline, such that negative age‐related changes in cognition were lesser in those with higher levels of mentally‐active SB. Additionally, follow‐up studies of Ringin et al. [68, 69] indicate that cognitive function may affect subsequent PA and SB, and increased passive SB correlates with poorer global cognition. In fact, the Ringin study [68] showed that both types of SB, alongside other health‐risk behaviors such as inadequate sleep duration and muscular strength (proxied by handgrip strength), were more meaningfully related to an index of global cognition than were other physiological markers of cardiometabolic disease, such as hbA1c (glycated hemoglobin) or CRP (C‐Reactive Protein). Whilst one might presume the temporal ordering of the effect to be of PA or SB influencing cognition, a further follow‐up study [69] showed that baseline cognitive performance in people with BD and health controls predicted higher levels of device‐measured sedentary time and moderate‐vigorous PA, and lower levels of light PA, 3–6 years later. Hence, the current sparse literature on the association of cognition with SB and PA is likely to be both bi‐directional and complex.

### 3.7 What Are the Risks of Prescribing Exercise in People With BD?

Concerns have been raised regarding whether exercise or increased PA may act as a precipitant for manic episodes in individuals with BD. A qualitative study [80] involving 26 individuals with BD type 1 reported subjective experiences suggesting that exercise could precipitate manic episodes. While such accounts are important for understanding patient perspectives and perceived risks, they remain anecdotal in nature and may be influenced by recall bias or individual symptom interpretations. In contrast, evidence from interventional studies using quantitative methods does not support a significant risk of mania induction through exercise. For instance, an open‐label, single‐arm study of Lafer et al. [84] monitored depressive and manic symptoms systematically at baseline and 2, 4, 8, and 12 weeks. Results showed no significant alterations in manic symptoms throughout the intervention. Similarly, several studies conducted by Sylvia et al. [90, 91, 92] and Li and colleagues [101] found no evidence that PA interventions led to treatment‐emergent mania. Collectively, this suggests that structured and supervised PA programs (exercise) appear safe with regard to mania risk in clinical trial contexts. However, more research is needed to clarify potential risks regarding excessive PA or exercise as a risk factor and indicator of worsening symptoms in people with mania.

Beyond mood symptoms, people with BD are at substantially elevated risk of presenting cardiometabolic conditions such as diabetes [105], hypertension, and metabolic syndrome [54], and cardiovascular diseases [115]. These comorbidities necessitate careful tailoring of exercise prescriptions. Recognizing this, the NExT Task Force emphasizes that PA and exercise interventions for individuals with BD should:

- Account for comorbid cardiometabolic conditions in the design and intensity of exercise programs;
- Ensure medical clearance and monitoring when needed, and
- Provide individualized adaptations that balance safety with therapeutic benefit, especially in patients at risk for mania;

In summary, while anecdotal evidence from one qualitative study suggests that exercise may precipitate manic symptoms, this has not been corroborated by other quantitative trials or observational studies. The current evidence supports the inclusion of PA or exercise in the management of BD, provided that cardiometabolic risks are considered and prescriptions are tailored accordingly. Nevertheless, further research is warranted to explore potential individual differences in mood reactivity to exercise and to ensure long‐term safety, particularly with respect to mood swings and manic relapses.

### 3.8 How can PA and SB be Assessed in BD?

PA and SB can be assessed using self‐reported measures (e.g., questionnaires) or objective methods (e.g., accelerometers). These approaches often yield different results, and understanding their respective strengths and limitations is critical for research and clinical practice. A summary of methods used in BD studies is presented in Table 3.

**TABLE 3:** Summary of the methods adopted to report physical activity or sedentary behavior in bipolar disorders.

**Subjective methods**
IPAQ [56, 58, 59, 64, 66, 68, 71, 72, 75, 77, 78, 107, 108, 112]
SIMPAQ [66, 110]
**Objective methods**
ActiGraph accelerometer [56, 60, 66]
Actiheart accelerometer [58]
Actiwatch accelerometer [63, 65, 70, 74, 79, 148]
Axivity accelerometer [81]
GENEActiv accelerometer [55]
Sensewear Armband [77]
Respironics and Actiwatch Score; Philips Respironics [65, 79]
Smartphone [71]

Abbreviations: IPAQ, International Physical Activity Questionnaire; SIMPAQ, Simple Physical Activity Questionnaire.

To date, most studies examining PA and SB have relied on self‐report instruments. Fourteen studies used the International Physical Activity Questionnaire (IPAQ) [56, 58, 59, 64, 66, 68, 71, 72, 75, 77, 78, 107, 108, 112], which remains the most widely employed tool in the general population. Two studies employed the Simple Physical Activity Questionnaire (SIMPAQ) [66, 110], which was specifically developed for populations with mental illness. However, several studies [56, 58, 66, 77] reported marked discrepancies between self‐reported and objectively measured PA or SB levels in BD.

For example, Nascimento et al. [56] compared the IPAQ with ActiGraph accelerometers and found significant differences between subjective reporting vs. objective measurement in moderate‐intensity PA (155 min vs. 25 min), moderate‐vigorous intensity PA (157 min vs. 50 min), and SB (480 min vs. 180 min). Freyberg et al. [58] reported similar findings when comparing the IPAQ with Actiheart accelerometers. Vancampfort et al. [77] compared IPAQ and Sensewear wristband accelerometers and found similar results for moderate‐intensity PA. IPAQ recorded 145 min, while Sensewear armband accelerometers recorded 168 min. Even with the similar time of moderate‐intensity PA, Vancampfort et al. observed closer agreement, but still noticed a difference of 40% in total energy expenditure (kcal/day) with the IPAQ underestimating caloric expenditure. Monteiro et al. [66] compared SIMPAQ with ActiGraph accelerometers and found discrepancies for moderate‐vigorous intensity (224 min vs. 137 min, respectively), and SB (325 min vs. 377 min, respectively).

These differences likely reflect social desirability bias and memory bias, both of which may be amplified in BD perhaps due to cognitive and emotional regulation impairments often associated with the disorder [121]. In samples with mental disorders, a meta‐analysis by de Oliveira Tavares et al. [123], found that the test–retest reliability of self‐reported questionnaires for measuring PA was moderate for intensity‐specific PA (*r* = 0.6) and strong (*r* = 0.8) for total PA. However, correlations of self‐reported questionnaires with objective measures were weaker for moderate (*r* = 0.2) than for vigorous PA (*r* = 0.3) and for total PA (*r* = 0.5).

The SIMPAQ, designed through a multidisciplinary collaboration to assess PA and SB in people living with mental illness [110], has been tested in 23 countries, including 78 participants with BD. Correlations with accelerometry were modest for PA (rho = 0.23—*p* < 0.04) and low for SB (rho = 0.08—*p* = 0.47) [110]. These findings highlight the challenges of accurately capturing activity patterns in this population.

Objective accelerometers, including devices like ActiGraph, Actiheart, and Sensewear armbands, provide precise data on PA intensity, duration, and frequency, as well as detailed SB patterns. These tools are less prone to subjective biases and are particularly valuable for capturing psychomotor variability, a characteristic feature of BD. Moreover, the accelerometer has difficulties distinguishing between running on a treadmill and strength training with weights, with light PA [149], as not permitted in different domains (leisure time activities, occupational activities, and transport/commuting activities). Additionally, accelerometers are costly, require technical expertise for deployment and analysis, and may fail to detect upper‐body movements or specific PA domains, limiting their ecological validity. Adherence can also be challenging in BD due to mood fluctuations affecting device adherence.

Both approaches (subjective and objective) provide complementary but imperfect insights. Questionnaires offer contextual richness but lack precision, whereas accelerometers deliver objective data but miss qualitative domains. Short questionnaires such as SIMPAQ can be helpful as screening tools and identifying those that may be at risk of low PA. Combining self‐reported and objective measures, such as integrating SIMPAQ with ActiGraph data, can enhance accuracy, comprehensiveness, and ecological validity by triangulating PA patterns while accounting for BD‐specific factors like mood state variability. Researchers should select methods based on study objectives, resources, and the need to balance feasibility with robustness, particularly in BD populations where behavioral fluctuations are prevalent.

### 3.9 Is Physical Inactivity an Environmental Risk Factor for BD? Is There a Protective Relationship Between PA and the Risk for Onset of BD?

The evidence on whether PA may protect against the onset of BD is mixed and remains inconclusive, drawing largely on prospective and Mendelian randomization studies.

Findings from prospective cohorts point in different directions. For example, a cohort study [111] of 2548 adolescents and young adults reported that those engaging in regular PA at baseline had a 10.3‐fold higher incidence of BD at a 4 year follow‐up. However, this result is difficult to interpret given the very small number of incident BD cases (*n* = 18) participants, emphasizing the need for replication in larger cohorts [111]. In contrast, the largest observational study to date [73], examined 196,685 participants in a long‐distance cross‐country ski race and compared them with 197,684 age and sex‐matched members of the general population. This study found a lower incidence of newly diagnosed BD favoring long‐distance cross‐country ski racers (adjusted hazard ratio, HR = 0.48). This finding suggests a potential protective association, though the unique nature of the activity (long‐distance skiing) limits generalizability.

Mendelian randomization studies which use genetic variants as proxies for exposures have produced similarly inconsistent results. Early work, such as Sun et al. [81], using accelerometer‐derived PA from the UK Biobank BD GWAS data from the Psychiatric Genomics Consortium, suggested that higher objectively measured overall PA was causally associated with a substantially lower risk of having BD. Notably, this protective effect was not observed when PA was based on self‐report, underscoring the importance of objective measurement. In contrast, more recent and larger‐scale bidirectional Mendelian randomization analyses, such as Iob et al. [21], failed to find robust evidence that higher PA causally reduces BD risk. Instead, they reported associations in the reverse direction, where genetic liability to BD was linked to higher objectively measured moderate PA and reduced SB time. The study of Monteiro et al. [66] showed differences in moderate‐vigorous PA time across BD phases, where 125 min/week was identified in participants of a euthymic mood state, a total of 102 min/week during a depressive episode, and a total of 206 min/week during a manic episode. These findings may reflect behavioral changes during manic and hypomanic states.

Further nuance arises from a broader Mendelian randomization study on SB and psychiatric disorders. While primarily focused on depression, this study reported unexpected results for BD, specifically, that greater leisure‐time television watching was associated with a lower risk of BD [19]. This counterintuitive finding underscores the complexity of disentangling causality in behavioral‐psychiatric links. Several factors can influence the outcomes, including the nature of the PA measure (objective vs. self‐report PA, specific activity types) and risk of measurement error, potential for weak genetic instruments, outcome GWAS power (inclusion of limited genome‐wide significant SNPs associated with the exposures of interest as PA, which could affect the power of the instruments), the potential for pleiotropy (where a single gene influences multiple, often unrelated traits), and the confounding variables considered (such as body mass index, educational attainment, and intelligence). All these factors can alter the results, and these considerations should be considered in interpreting the findings of these studies. Future studies could benefit from incorporating brief, multidimensional assessment instruments of lifestyle since many studies did not control other lifestyle behaviors, particularly diet/nutrition and restorative sleep [150].

Collectively, the current data from prospective studies do not yet provide definitive evidence that increased PA reduces or increases the risk of BD. Instead, the findings point to a more complex, potentially bidirectional relationship that may be contingent on activity type, illness phase, and methodological approach. Clarifying these associations will require larger, more representative longitudinal studies, coupled with refined behavioral measures.

## 4 Conclusion

**FIGURE 1:**
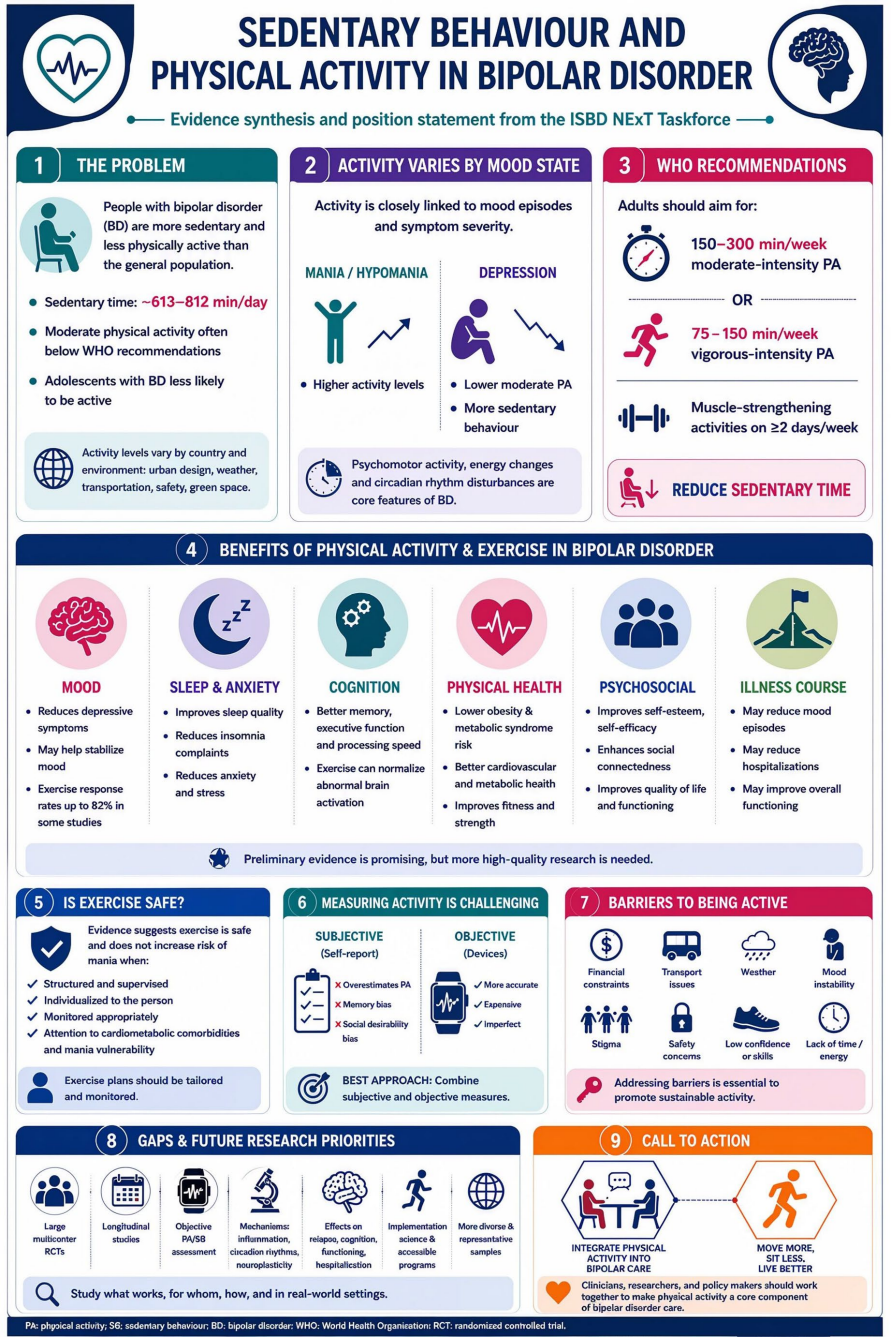
Infographic regarding sedentary behavior and physical activity in BD.

BD is associated with markedly elevated rates of medical comorbidities, including metabolic syndrome, cardiovascular disease, diabetes, and obesity, which collectively drive premature mortality. Population‐level studies, alongside recommendations from authoritative task forces and institutions, underscore the potential of PA and exercise programs to mitigate these health disparities. Beyond their cardiometabolic benefits, emerging preliminary evidence suggests that PA and exercise may confer antidepressant and pro‐cognitive effects in individuals with BD, potentially enhancing mood stability, functional outcomes, and quality of life (Figure 1).

Nevertheless, the BD‐specific literature on PA and SB remains fragmented, methodologically heterogeneous, and constrained by small sample sizes, reliance on self‐reported measures, and limited longitudinal designs [151, 152]. These shortcomings preclude definitive conclusions on causal pathways, optimal intervention parameters (e.g., intensity, duration, and modality), and long‐term efficacy in diverse subpopulations, such as those with varying illness severity, comorbidities, or socioeconomic backgrounds. To address these gaps and fortify the evidence base, large‐scale, multicenter randomized controlled trials are imperative. Such studies should rigorously evaluate whether PA, SB and exercise exert protective effects against BD onset, modulate illness trajectory (e.g., reducing relapse rates or hospitalizations), and yield antidepressant, pro‐cognitive, and anti‐inflammatory benefits through mechanistic investigations, including biomarkers of neuroinflammation, circadian rhythm regulation, and neuroplasticity. Multicenter approaches are essential to account for geographical, cultural, and socioeconomic variations that currently confound findings, ensuring generalizability across global populations.

Additional research priorities include implementation science to dismantle barriers to PA engagement, particularly for underserved groups facing socioeconomic, structural, or motivational obstacles. This entails developing affordable, scalable, community‐based interventions that minimize equipment dependence and incorporate behavioral strategies to enhance adherence, thereby alleviating the psychological and other health burdens associated with BD [117]. The refinement and validation of assessment tools tailored to BD, integrating self‐reported instruments (e.g., validated questionnaires) with objective measures (e.g., accelerometers and smartphone apps) to capture nuanced activity patterns, including temporal variability linked to pathological mood states should also be a priority for future studies.

In summary, the Nutrition and Exercise Task Force (NExT) advocates for the integration of PA, SB, and exercise into global BD management protocols by clinicians, urges policymakers and funders to prioritize inclusive programs, and calls upon researchers to pursue rigorous, collaborative inquiries. These efforts hold promise for transforming BD care, bridging the gap between lifestyle interventions and improved physical and mental health outcomes.

## Conflicts of Interest

B.S. is supported by an NIHR Advanced Fellowship (NIHR301206). B.S. is on the Editorial Board of the Journal of Physical Activity and Health, Aging Research Reviews, Mental Health and Physical Activity, The Journal of Evidence Based Medicine, and The Brazilian Journal of Psychiatry. Brendon has received honorarium from a co‐edited book on exercise and mental illness (Elsevier), an associated education course and unrelated advisory work from ASICS and FitXR LTD; A.G.‐P. has received grants and served as consultant, advisor or CME speaker for the following entities: Janssen‐Cilag, Lundbeck, Otsuka, Alter, Angelini, Novartis, Rovi, Takeda, the Spanish Ministry of Science and Innovation (CIBERSAM), the Ministry of Science (Carlos III Institute), the Basque Government, and the European Framework Program of Research; E.V. has received grants and served as consultant, advisor or CME speaker for the following entities: AB‐Biotics, AbbVie, Adamed, Angelini, Biogen, Biohaven, Boehringer‐Ingelheim, Celon Pharma, Compass, Dainippon Sumitomo Pharma, Ethypharm, Ferrer, Gedeon Richter, GH Research, Glaxo‐Smith Kline, HMNC, Idorsia, Janssen, Lundbeck, Medincell, Merck, Novartis, Orion Corporation, Organon, Otsuka, Roche, Rovi, Sage, Sanofi‐Aventis, Sunovion, Takeda, and Viatris, outside the submitted work; F.A.G. reports consultant/speaker fees from AbbVie, Lundbeck, Otsuka, and Johnson & Johnson, outside the submitted work; L.N.Y. reports consultant/speaker fees from AbbVie, Alkermes, Intracellular Therapies, LivaNova, Lupin, Neurotorium, Newron, Otsuka, Puretech, Sumitomo Pharma, and Xenon, and grants from AbbVie, CIHR, and Sumitomo Pharma, outside the submitted work, over the last 3 years; L.S. has received grant funding from PCORI, NIH, HRSA, Tiny Blue Dot Foundation, and AFSP. She has also received funding as a member of the Milken Institute's Scientific Advisory Board as well as royalties form New Harbinger for her two, published books on bipolar disorder; M.G.‐R. receives research support from the Mexican Ministry of Science, Humanities, Technology, and Innovation (Secihti); M.S. has received honoraria/has been a consultant for AbbVie, Angelini, Bausch Health, Boehringer Ingelheim, Lundbeck, Otsuka, Teva; M.V. has received honoraria from Eli Lilly, Lundbeck Pharma and Johson & Johnson; R.M. has received consulting and speaking honoraria from AbbVie, Biogen, Lallemand, Lundbeck, and Otsuka, and research grants from Brain Canada, CAN‐BIND, CIHR and OBI; T.V.R. has received travel funding from the Lundbeck Foundation/Neurotorium and grants from the NHMRC, Rebecca L Cooper Foundation, Helen McPherson Smith Trust, Milken Baszucki Brain Research Fund, Australian Rotary Health, University of Melbourne, Barbara Dicker Brain Sciences Foundation and the Society for Mental Health Research—all unrelated to this work; M.B.: Grant funding: Wellcome Trust, Medical Research Future Fund, Victorian Government Department of Jobs, Precincts and Regions, Janssen Lundbeckfonden Copenhagen, St. Biopharma, Milken Baszucki Brain Research Fund, Stanley Medical Research Institute, Danmarks Frie Forskningsfond Psykiatrisk Center Kovenhavn, Patient‐Centered Outcomes Research Institute (PCORI), Australian Eating Disorders Research and Translation Centre AEDRTC, USA Department of Defense Office of the Congressionally Directed Medical Research Programs (CDMRP), Equity Trustees Limited. Advisory boards: Janssen, Otsuka, St Biopharma, Actinogen, Servier—all unrelated to this work; W.M. has previously received funding from the Cancer Council Queensland and university grants/fellowships from La Trobe University, Deakin University, University of Queensland, and Bond University. W.M. has received funding and/or has attended events funded by Cobram Estate Pty. Ltd. and Bega Dairy and Drinks Pty Ltd. W.M. has received travel funding from Nutrition Society of Australia. Wolfgang has received consultancy funding from Nutrition Research Australia and ParachuteBH. W.M. has received speaker honoraria from VitaFoods, The Cancer Council Queensland and the Princess Alexandra Research Foundation; F.S. is Associate Editor of the Mental Health and Physical Activity journal and the Jornal Brasileiro de Psiquiatria and is on the Board of The Brazilian Journal of Psychiatry, and Trends in Psychiatry and Psychotherapy. F.S. has received honorarium from two co‐edited books on exercise and mental health (MANOLE).

## Data Availability

The data that support the findings of this study are available from the corresponding author upon reasonable request.
